# Patients with unstable angina and myocardial infarction expose remote VSMC phenotype switch and alteration in the proliferation of smooth muscular cell in the aortic wall

**DOI:** 10.1186/1749-8090-10-S1-A238

**Published:** 2015-12-16

**Authors:** Chin Cheng Woo, Tang Zhiqun, Yang Sun Chan, Xiao Yun Lin, Richie Soong, Chuen Neng Lee, Vladimir Kuznetsov, Sorokin Vitaly

**Affiliations:** 1 Department of Surgery, Yong Loo Lin School of Medicine, National University of Singapore, Singapore; 2 Bioinformatics Institute (BII), Agency for Science, Technology and Research (A*STAR), Singapore; 3 Genome Institute of Singapore (GIS), Agency for Science, Technology and Research (A*STAR), Singapore; 4 Department of Cardiac, Thoracic and Vascular Surgery, National University Heart Centre, National University Health System, Singapore; 5 Cancer Science Institute of Singapore, National University of Singapore, Singapore

## Background/Introduction

Vascular smooth muscular cell (VSMC) involve in vessel tone regulation and endothelial cell function. It is known and critical player in pathological conditions including atherosclerosis. VSMC is dynamic structure and response to different stimuli. MiRNAs molecules are important regulatory mechanism to promote/suppress VSMC proliferation and phenotype switch.

## Aims/Objectives

Aim: We hypothesized that patients with acute myocardial infarction have altered VSMC transcriptome affecting cell phenotype and proliferation.

## Method

Methods: Aortic wall tissue obtained during coronary artery bypass surgery underwent laser dissection of VSMC. Frozen aortic sections were stained with Arcturus LCM kit to identify cells of interest follow by cell collection and tRNA extraction. The transcriptome profile will be performed using Affymetrix GeneChip^® ^Human Genome U133 Plus 2.0 Array. The differently expressed mRNA/miRNA data were validated on independent cohort of patients with RT-PCR method assay.

## Results

Result: We identify nine miRNA differentially expressed in patients with acute myocardial infarction, with four of them related to VSMC proliferation and differentiation. The most prominent down regulated miRNA was known regulator of VSMC phenotype miR-143. Among up regulated miRNA, miR-486-5p and miR-29a-3p reported before as a potent regulator of VSMC function. Pairwise analysis with mRNA data from the same patients reveal reciprocal and statistically significant changes in the phenotype and proliferation related mRNA.

## Discussion/Conclusion

Conclusion: In patients with acute myocardial infarction we observe remote phenotype and proliferation shift in the aortic wall VSMC. Switch of contractile phenotype and proliferation alteration might play compensatory role in patient with unstable plaque and myocardial infarction.

